# Potential of Exogenous Treatment with Dehydroascorbate to Control Root-knot Nematode Infection in Rice

**DOI:** 10.1186/s12284-023-00644-1

**Published:** 2023-06-29

**Authors:** Satish Namdeo Chavan, Farzana Haque Tumpa, Md. Atiqur Rahman Khokon, Tina Kyndt

**Affiliations:** 1 Department of Biotechnology, Faculty of Bioscience Engineering, Ghent University, Proeftuinstraat 86 N1, Ghent, 9000 Belgium; 2 ICAR-Indian Institute of Rice Research, Rajendranagar, Hyderabad, 500030 India; 3 Department of Plant Pathology, Bangladesh Agricultural University, Mymensingh, 2202 Bangladesh

**Keywords:** Dehydroascorbate, Induced resistance, Integrated pest management, *Meloidogyne graminicola*, Nematicidal, Rice

## Abstract

Induced resistance (IR) is a unique physiological state characterized by reduced plant susceptibility to (a)biotic stress. Our previous studies showed that exogenous foliar application of dehydroascorbate (DHA), the oxidized form of ascorbic acid, induces systemic resistance against root-knot nematode *Meloidogyne graminicola* in rice. In the present study, the potential of DHA in protecting rice plants against *M. graminicola* was evaluated in lab, pot, and field studies. In an experiment where the interval between foliar treatment and inoculation was varied, 20 mM DHA was found to protect rice plants from *M. graminicola* for at least 14 days. Pot and field studies confirmed that 10 or 20 mM DHA are highly effective in reducing gall formation and led to a significant increase in rice seed yield. A half dose of DHA (10 mM) combined with another IR-stimulus - piperonylic acid (PA) 300 µM - was at par with DHA 20 mM, leading to reductions in gall formation of more than 80%. In in vitro bioassays, DHA was found to be highly nematicidal to the second-stage juveniles of *M. graminicola*, with more than 90% mortality within 3 h of exposure to 10 or 20 mM concentrations. While seed treatment had no effect, root drenching or root dipping was also effective in reducing rice susceptibility to *M. graminicola*, next to foliar treatment. As a dual-action compound with extended protection and ease of application, DHA has great potential for effective nematode management in rice.

**Supplementary Information:**

The online version contains supplementary material available at 10.1186/s12284-023-00644-1.

## Key Message

• This research demonstrates the use of dehydroascorbate as a sustainable approach for *M. graminicola* management in rice.

• DHA protects the rice plants from *M. graminicola* for at least up to 14 days after its application.

• Pot and field studies showed the great potential of DHA in reducing nematode infection with a concomitant increase in seed yield.

• DHA was found compatible with another IR stimulus PA in reducing rice susceptibility to *M. graminicola*.

• In addition to reducing rice susceptibility, DHA was found highly nematicidal to *M. graminicola*.

• DHA is effective in rice as a foliar treatment, root drench, or root dip treatment against *M. graminicola*.

**Supplementary Information:**

The online version contains supplementary material available at 10.1186/s12284-023-00644-1.

## Introduction

Rice is one of the most widely cultivated and strategic crops globally for food and nutrition security (Fairhurst and Dobermann 2002; FAO 2021) but is prone to several biotic and abiotic stresses. Root-knot nematode (RKN) *Meloidogyne graminicola* is one of the economically most important plant-parasitic nematode problems in all types of rice cultivation (Dutta et al. 2012; Gaur 2021, 2023; Mantelin et al. 2017; Prasad et al. 2010; Ravindra et al. 2017; Rusinque et al. 2021). It causes severe qualitative and quantitative losses in upland, lowland, and deep-water rice. Significant damage also occurs in nursery beds since young seedlings are highly susceptible to *M. graminicola* (Dangal et al. 2009; Gaur 2021). The second-stage juveniles (J2s) of *M. graminicola* infect the roots of rice plants. The infected root tips become swollen and produce characteristic hooked galls affecting overall root growth. The affected plants look pale yellow with stunted growth and reduced tillers and panicles. As a result, *M. graminicola* can cause up to 87% yield loss in rice production (Mantelin et al. 2017). J2s of *M. graminicola* can survive during the off-season and remain viable in soil without a host plant for up to five months (Bridge and Page 1982; Soomro 1989). The large-scale introduction of water-saving rice production systems, such as direct wet seeding, intermittent irrigation, cultivation on raised beds, and aerobic rice techniques are favoring the development of high populations of *M. graminicola*, drastically increasing its economic significance (Waele and Elsen 2007; Mantelin et al. 2017; Rusinque et al. 2021). Its short life cycle and broad host range, including many weed species common in rice fields, make this species difficult to control (Waele and Elsen 2007; Mantelin et al. 2017; Ravindra et al. 2017).

Induced resistance (IR) is a promising novel approach in the search for environmentally-friendly pest and disease management strategies (Walters and Fountaine 2009; Walters et al. 2013; Yassin et al. 2021). IR refers to a physiological state of a plant induced by exposure to an external stimulus and characterized by reduced susceptibility to (a)biotic stresses (De Kesel et al. 2021). IR stimuli include natural or chemical compounds, beneficial microbes, and various (a)biotic stresses (Conrath et al. 2006; Mauch-Mani et al. 2017; Somasekhar 2008). Some IR stimuli are commercially available, such as acibenzolar-S-methyl (ASM) (Romero et al. 2001), probenazole (Iwata et al. 2004; Yoshioka et al. 2001), Chitosan (Fitza et al. 2013; Tumpa et al. 2017, 2018; Tumpa and Khokon 2020), and COS-OGA, a combination of chito-oligosaccharides and oligogalacturonides (Singh et al. 2019a; Van Aubel et al. 2014).

IR involves direct activation of defence responses upon contact with the stimulus and/or defence priming where the immune responses are potentiated to react robustly to stress exposure (Conrath et al. 2006; De Kesel et al. 2021; Mauch-Mani et al. 2017). In many cases, IR is based on a combination of this direct induction and defence priming and involves activation of local and/or systemic resistance (Chavan et al. 2022; De Kesel et al. 2021; Desmedt et al. 2021). The defence mechanisms involved in IR include the oxidative burst (Chavan et al. 2022; Desmedt et al. 2021; Wojtaszek 1997), activation of plant hormone pathways (Denancé et al. 2013; Martínez-Medina et al. 2017), phenylpropanoid pathway disturbance (Singh et al. 2019a, 2021), accumulation of proteins with anti-pathogen activity (van Loon et al. 2006), production of phytoalexins (Desmedt et al. 2022b), and cell wall reinforcement (Malinovsky et al. 2014; Veronico et al. 2018). The resulting response tends to be broad-spectrum and can be long-lasting but is rarely complete, with most stimuli reducing disease severity by between 20 and 85% (Walters et al. 2013).

Integrated pest management (IPM) is a strategy for combating plant pests and diseases, using all available environmentally friendly approaches while minimizing the use of chemical pesticides (Ehler 2006). IR fits well into IPM as activation of innate plant immunity could replace or reduce the pesticide dosage (Yassin et al. 2021). The efficacy of IR stimuli can be improved by combining them with other IR agents (Reuveni et al. 2001; Walters et al. 2011), bio-stimulants (Pereira et al. 2021), biocontrol agents (Abd El-Rahman and Mohamed 2014; De Jong et al. 2019; Singh et al. 2019b; Yi et al. 2013; Zehra et al. 2017), or pesticides (Baider and Cohen 2003; Liljeroth et al. 2010; Percival and Graham 2021; Reuveni et al. 2001; Sharma et al. 2011). Besides combining IR agents with chemical pesticides, another strategy to improve their efficacy is identifying compounds combining biocidal and IR activity (Schouteden et al. 2017; Yassin et al. 2021) or by devising a proper method of application (Molinari 2016; Pankaj et al. 2013). Activation of plant defence systems has been described to be associated with a fitness cost, as it requires energy and resources (Walters et al. 2013). However, the extent of the fitness penalty differs largely between stimuli and is dependent on the growth environment (Van Hulten et al. 2006; Walters and Heil 2007). Hence, potential changes in plant growth and development should be monitored upon IR activation (Yassin et al. 2021).

Compounds, such as ethephon, methyl jasmonate (MeJA), salicylic acid (SA) analogue benzothiadiazole (BTH) (Nahar et al. 2011), beta-aminobutyric acid (BABA) (Ji et al. 2015), thiamine (Huang et al. 2016), silicon (Zhan et al. 2018), COS-OGA (Singh et al. 2019a), ascorbate oxidase (AO) (Singh et al. 2020a, b, 2021), and phenylpropanoid pathway inhibitor piperonylic acid (PA) (Desmedt et al. 2021) are known to induce resistance against plant-parasitic nematodes. Recently, we showed that the exogenous foliar application of dehydroascorbate (DHA), the oxidized form of ascorbic acid (AsA), activates systemic rice resistance against RKN *M. graminicola* (Chavan et al. 2022). DHA-IR activation leads to reduced nematode penetration and development and this was mediated via the increased production of reactive oxygen species (ROS), salicylic acid (SA) signaling (Chavan et al. 2022), and diterpenoid phytoalexins in the rice plants (Desmedt et al. 2022b). In the present work, we evaluated the potential of DHA for nematode control in lab, pot and field studies and aimed to increase its efficacy by combining DHA treatments with PA and using alternative application methods. The nematicidal property of DHA was evaluated against the second-stage juveniles of *M. graminicola*. The obtained results will be useful for devising a better technique for the management of nematode problems in rice cultivation.

## Materials and Methods

### Chemical Treatment

Rice plants were treated with 10 or 20 mM of DHA (L-dehydroascorbic acid, Sigma-Aldrich, Cat. No. 261,556) (Chavan et al. 2022) and/or 300 µM PA (piperonylic acid, Sigma-Aldrich, Cat. No. P49805) (Desmedt et al. 2021). The chosen 20 mM concentration of DHA was previously optimized for best efficacy against *M. graminicola* and lack of phytotoxicity (Chavan et al. 2022). In order to potentially reduce the dose of DHA, the half dose, i.e., 10 mM was used while combining it with PA. Since PA was dissolved in DMSO, an additional mock treatment with only DMSO was included. Each plant was treated with 6.25 ml solution or distilled water containing 0.02% (v/v) of Tween20 (Sigma-Aldrich, Cat. No. P1379) for efficient spread and uptake of chemicals. Plants were treated by spraying the above-ground parts using a hand automizer sprayer.

### Lab Nematode Infection Assays in the Plant Growth Room

Seeds of rice (*Oryza sativa*) variety Nipponbare (GSOR-100; USDA) were germinated in the dark for 4 days at 30 °C and were transferred to polyvinyl chloride (PVC) tubes (diameter 3 cm, length 18 cm) containing SAP substrate (sand mixed with Absorbent Polymer AquaPerla; DCM) (Reversat et al. 1999). Plants were further grown in a rice growth room at 26 °C under a 12 h/12 h light/dark regime (Supplementary material Fig. S1). Plants were irrigated three times a week with 10 ml of Hoagland’s solution each time (Hoagland and Arnon 1950).

A pure culture of RKN *M. graminicola* was originally obtained from the Philippines (kindly provided by Professor Dirk De Waele, KU Leuven; Batangas population) and maintained on susceptible grass (*Echinochloa crus-galli*). Freshly hatched second-stage juveniles (J2s) were used for plant inoculation. Two-week-old rice plants were inoculated with 250 J2s per plant or mock-inoculated with water at 1 day post-treatment (DPT).

In an experiment to evaluate the longevity of the DHA control effect, two-week-old rice plants were treated with DHA 20 mM and nematodes were inoculated at different time points after treatment: 1, 3, 7, or 14 DPT.

In an experiment to evaluate different methods of DHA applications the efficacy of foliar application, root drench, root dip, and seed treatment were evaluated. For seed treatment, seeds were incubated in 20 mM DHA solution containing 2% carboxymethyl cellulose for 20 min and then transferred to wet filter paper for germination. For foliar spray, two-week-old plants were treated as described above. For root drench, 6.25 ml of DHA 20 mM or distilled water (control) was drenched on the SAP substrate of each plant. For root dip treatment, two-week old plants were uprooted carefully, roots were washed gently and dipped into 20 mM DHA solution or in distilled water (control) for 20 min. Nematodes were inoculated 24 h after foliar application, root drench or root dip treatment. In the case of seed treatment, nematodes were inoculated two-weeks after seedling transplantation into the SAP tubes.

Plant susceptibility was assessed two weeks post nematode inoculation by counting galls, total nematodes, and egg-laying females in the roots using the acid fuchsin staining technique (Byrd et al. 1983). All nematode infection assays were repeated at least twice, each time including eight to twelve plants per treatment.

### Pot Experiments in the Net-house

The effect of DHA and PA on plant susceptibility to *M. graminicola* and rice growth and yield was evaluated in a pot experiment in the net house of Professor Golam Ali Fakir Seed Pathology Centre, Department of Plant Pathology, Bangladesh Agricultural University, Mymensingh, Bangladesh (Supplementary material Fig. S2). The experiment was conducted during the Boro season (January – April 2022). Plastic pots of 15 kg volume were laid out in a completely randomized design (CRD) with eight replications per treatment. The pots and soil used for the experiment were pre-fumigated with 5% formaldehyde to kill potential pathogens (Al-Khatib et al. 2017). Well-decomposed compost was mixed with the soil prepared at the rate of 10 t/ha and then triple super phosphate, muriate of potash, gypsum, ZnSO_4_ and Boric acid were added at the rate of 1, 0.4, 0.06, 0.06 and 0.03 g per pot respectively at the time of planting (BRRI 2017). Additionally, urea (1.8 g/pot) was added in three splits at the seedling, tillering, and panicle initiation stage (Lyu et al. 2021). Seeds of rice variety BRRIdhan 28 were germinated first in separate nematode-free trays, after which one month old seedlings were transplanted to new nematode-free pots for the experiment. The first treatments were done 15 days after transplanting (DAT), followed by six spraying at 10 days intervals. Nematodes were inoculated at 250 J2s per plant or mock-inoculated with water at 1 day after the first treatment by making small holes around the plants. The pots were irrigated once a day. Weed control was achieved using hand weeding and recommended agronomic practices were followed throughout the crop growth.

The pot study was repeated during Aman season (August – November 2022) using another widely grown rice cultivar BRRIdhan 49 in Bangladesh, with the same set-up.

Plants were treated with DHA and PA alone, or in combination, or with appropriate control treatments. Six treatments were included: T_1_-Untreated control, T_2_-DHA 10 mM, T_3_-DHA 20 mM, T_4_-PA 300 µM, T_5_-DHA 10 mM + PA 300 µM, T_6_-DMSO 300 µM. The chemicals were applied as mentioned above.

### Field Experiment

A field experiment was conducted in the naturally nematode-infested field located at Central Farming System research farm, Bangladesh Agriculture University, Mymensingh, Bangladesh (Supplementary material Fig. S3). The experimental site was located at 24°75’ N latitude and 90°50’ E longitude at an elevation of 18 m above the mean sea level. The experimental area was characterized by non-calcareous dark grey floodplain soil belonging to the Sonatola Soil Series under the Old Brahmaputra Floodplain, Agro-Ecological Zone 9 (Shil et al. 2016). The soil of the experimental field was more or less neutral in reaction with a pH value of 6.8, low in organic matter and fertility level. The land type was medium high with sandy loam in texture. The climate of the locality is tropical in nature and is characterized by high temperatures and heavy rainfall during the Kharif season (April to September) and scanty rainfall associated with moderately low temperature during Rabi season (October to March). The experiment was carried out during the Boro season (January-April 2022). The pre-trial nematode population (Pi) was assessed by following the method of Viglierchio and Schmitt (1983).

Seedlings of cultivar BRRIdhan28 were grown in a raised nursery beds in nematode-free soil. One month old seedlings were transplanted to the main field. The size of each experimental plot was 1 m × 1 m and laid out in a randomized complete block design with eight replications per treatment. The field was prepared by deep ploughing followed by harrowing. A fine puddled structure was achieved by a tractor-drawn plough with planking after applying ample irrigation. Well-decomposed compost (1 kg/m^2^) was applied in the field before puddling. The recommended dose of triple super phosphate, muriate of potash, gypsum, ZnSO_4_ and Boric acid were mixed well with the soil at the rate of 18, 15, 1, 1 and 1 g/m^2^, respectively, according to the Adhunik Dhaner Chas Handbook (BRRI 2017). Irrigation and draining out of excess water in the experimental plots was done whenever needed (depending on rainfall). Weed control was achieved using hand weeding and recommended agronomic practices were followed throughout the crop growth. The same treatments as described above in the pot experiment were included.

### Observations and Recording Data for Pot and Field Experiments

The plant height was recorded at 10 day intervals during the entire growth of the rice plants by measuring from the base of the plant to the tip of the tallest leaf. Data on yield and other growth parameters, viz., number of tillers, number of panicles, length of panicles, number of grains per panicle, straw yield, seed yield, 1000 grain weight, were recorded at the time of harvesting (120 DAT). After harvest, the plants were uprooted carefully, and roots were processed using the acid fuchsin staining technique (Byrd et al. 1983) to count galls for evaluating plant susceptibility to *M. graminicola*.

### Nematicidal Assay

In an *in-vitro* bioassay, different DHA concentrations ranging from 1.25 to 20 mM were tested against J2s of *M. graminicola*. In each well of a 12-well cell culture plate (Greiner Bio-One, Cat. No. 665 − 180), around 100 J2s were incubated in 1 ml solutions with different concentrations of DHA: 1.25, 2.5, 5, 10, and 20 mM. Distilled water was used as a negative control. Four replications were used per treatment. Observations on nematode mortality were recorded 3, 6, 12, 24, 48, and 72 h after incubation using a stereo microscope. Nematode mortality was assessed by probing the nematodes with a fine needle and incubating immobile nematodes in fresh distilled water for 24 h. Dead nematodes become straight and immobile and do not move upon probing with fine needles (Supplementary material Fig. S4).

The acute toxicity of chemical substances against test organisms is often presented with their LC_50_/LD_50_ values (ECETOC 1984). To determine the potential toxicity of DHA against J2s of *M. graminicola*, an LC_50_ analysis was done. The LC_50_ (median lethal concentration) is the lethal concentration of a pesticide/substance that kills 50% of a sample population in a given time period (Burgess et al. 2020). DHA caused strong nematode mortality at 10 and 20 mM concentrations within 3 h of exposure (See further, Fig. 5). Hence, in order to determine the correct dose response and to narrow down the confidence interval (95% fiducial limits), we used a lower range of DHA concentrations, i.e., 1, 2, 3, 4, 5, and 6 mM for LC_50_ determination. The experiment was carried out in 12-well cell culture plates, as described above. Observations on nematode mortality were recorded at 6, 12, 24, 48, and 72 h after exposure, as described above.

### Statistical Analysis

Various statistical analyses (ANOVAs, post hoc tests, and Student’s t-test applied whenever appropriate, as indicated in the corresponding figure legends) were performed in SPSS Statistics 26.0 and R software (V.4.0.2.). The assumptions of normality and homogeneity of the data were checked and found to be fulfilled.

## Results

### Foliar DHA treatment Reduces Rice Susceptibility to ***M. graminicola*** and Protects the Plants for at Least 14 Days After Treatment

**Fig. 1:**
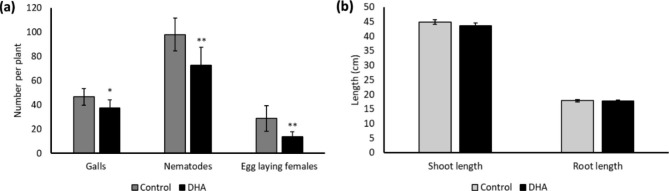
Effect of dehydroascorbate (DHA) on plant susceptibility to *Meloidogyne graminicola*. Two-week-old rice plants were treated with 20 mM DHA followed by nematode inoculation 250 J2s per plant 24 h post-treatment. Effect on (**a**) galls, total nematodes, and egg-laying females and (**b**) shoot and root lengths of rice plants recorded two-week post nematode inoculation. Bars on each column indicate SE from eight replicates. The experiment was independently repeated three times, providing confirmatory results. *Asterisks on error bar indicate statistically significant difference (Student’s t-test, * = p < 0.05, ** = p < 0.01)

Confirming previous observations reported in Chavan et al. (2022), foliar 20 mM DHA treatment in rice caused reduced *M. graminicola* infection (Fig. 1a). Number of galls, total nematodes, and egg-laying females were significantly reduced in 20 mM DHA-treated plants compared to control plants (Fig. 1a). The reduced number of egg-laying females upon DHA treatment shows that nematode reproduction is also affected (Fig. 1a). These results, combined with our detailed transcriptome analyses (Chavan et al. 2022) show that DHA-treatment induces plant resistance in rice against RKN *M. graminicola*. No negative effects on plant growth were observed upon DHA treatment (Fig. 1b).

**Fig. 2:**
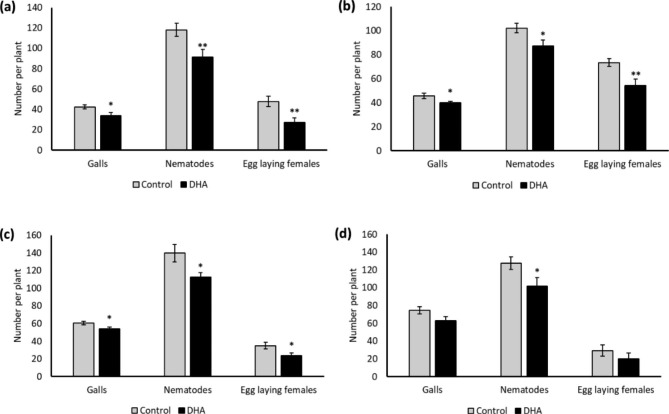
Longevity of dehydroascorbate (DHA)-induced resistance in protecting the rice plants from *Meloidogyne graminicola*. Two-week-old rice plants were treated with 20 mM DHA followed by nematode inoculation 250 J2s per plant (**a**) 1, (**b**) 3, (**c**) 7, and (**d**) 14 days post-treatment. Observations on galls, total nematodes, and egg-laying females were recorded two-week post nematode inoculation. Bars on each column indicate the SE from eight replicates. The experiment was independently repeated two times, providing confirmatory results. *Asterisks on error bar indicate statistically significant difference (Student’s t-test, * = p < 0.05, ** = p < 0.01)

To be able to design efficient management strategies for rice fields, it is important to know how long DHA protects the plants after its application. More specifically, this information is useful to select the number of sprays and duration between consecutive sprays during the growth season. A significant reduction in galls, nematodes, and egg-laying females was observed in the plants that were pretreated with 20 mM DHA up to 7 days before inoculation (Fig. 2). However, the effect is vanishing after 14 days, when only a reduction in the total number of nematodes was detected (Fig. 2). These results show that the IR effect induced upon DHA treatment lasts for at least 14 days after treatment.

### Efficacy of DHA in Pot Experiment in the Net House

**Fig. 3:**
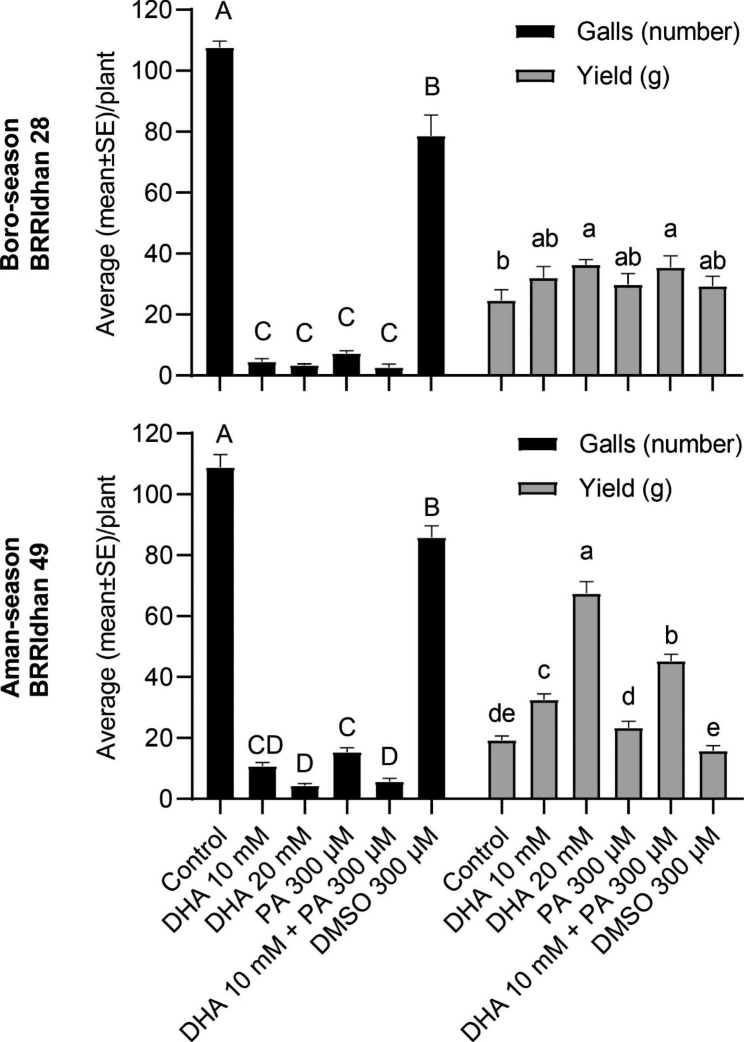
Effect of DHA and PA on rice susceptibility to *Meloidogyne graminicola* and seed yield. The experiment was conducted in pots in a net house using cultivar BRRIdhan 28 during the Boro season (January – April 2022; above). Plants were treated with DHA or PA alone or in combination as a foliar application or mock-treated with water or DMSO 15 days after transplanting (DAT) into the pots, followed by six spraying at 10 days intervals. Effect on galls and seed yield was recorded at the time of harvesting (120 DAT). The experiment was repeated during the Aman season (August – November 2022; below) using cultivar BRRIdhan 49. Bars on each column indicate SE from eight replicates. Different letters on error bars within a group indicate a statistically significant difference (DMRT; α = 0.05)

Based on the results of lab experiments, a pot study was conducted to evaluate the efficacy of foliar applications of DHA and in combination with PA under semi-natural conditions in Bangladesh, one of the major rice-growing countries. This experiment was done in the Boro season, using rice cultivar BRRIdhan 28, which is widely grown in Bangladesh. Based on the results obtained in the longevity experiment (Fig. 2), foliar treatment was repeated with 10 day-intervals throughout the growth season. In this experiment, a second IR stimulus was included: piperonylic acid (PA) that transiently inhibits the plant cinnamate-4-hydroxylase enzyme (Desmedt et al. 2021). The control treatment only receiving DMSO – the solvent used for PA dissolution – lead to minor reduction in gall formation (Fig. 3). Confirming and even surpassing our previous observations with single applications in the lab with cultivar Nipponbare (Fig. 1), repeated foliar DHA treatments (10 or 20 mM) in BRRIdhan 28 caused a very strong reduction in galls (95 and 96%) compared to the mock-treated plants (water) (Fig. 3). Similarly, the 300 µM PA application also caused 93% reduction in galls, validating our previously described lab results (Desmedt et al. 2021).

The combined application of two distinct IR stimuli, DHA and PA, led to increased plant protection from *M. graminicola* infection (Fig. 3). Indeed, the highest reduction in galls was observed in the combined application of DHA 10 mM and PA 300 µM (97.6%) compared to the mock-treated plants (Fig. 3).

A significant increase in yield was observed in plants treated with 20 mM DHA or receiving the combined application of DHA 10 mM and PA 300 µM (Fig. 3). An overall increase in plant height, number of tillers, panicles, number of grains per panicle, panicle length, total grains per panicle, filled/healthy grains per panicle, 1000 grain weight, and dry matter accumulation were observed in these treatments (Tables 1 and 2). These results indicate a positive effect of DHA on plant growth and yield.

**Table 1:** Effect of DHA and PA on growth of rice cv. BRRIdhan 28. The experiment was conducted in pots in a net house during the Boro season (January – April 2022). Plants were treated with DHA or PA alone or in combination as a foliar application or mock-treated with water or DMSO 15 days after transplanting (DAT) into the pots, followed by six spraying at 10 days intervals

Treatments	15 DAT	25 DAT	35 DAT	45 DAT	55 DAT	65 DAT	75 DAT	85 DAT	120 DAT
Control	20.88 ± 1.0 ab	30.00 ± 2.5 a	46.06 ± 3.1 a	53.50 ± 3.2 b	63.25 ± 2.3 a	75.38 ± 2.6 b	87.13 ± 4.2 b	98.88 ± 4.1 b	108.00 ± 5.4 a
DHA 10 mM	19.38 ± 1.2 ab	31.38 ± 1.4 a	49.38 ± 2.3 a	58.50 ± 3.1 ab	67.63 ± 2.8 a	81.50 ± 2.1 ab	96.13 ± 2.5 ab	110.00 ± 1.4 a	112.63 ± 1.3 a
DHA 20 mM	19.13 ± 0.8 b	34.94 ± 1.8 a	51.20 ± 1.9 a	62.75 ± 2.2 a	71.63 ± 2.0 a	84.88 ± 2.0 a	100.31 ± 4.5 a	108.63 ± 4.4 ab	116.38 ± 4.2 a
PA 300 µM	20.50 ± 1.3 ab	31.25 ± 1.2 a	47.00 ± 0.9 a	58.13 ± 1.2 ab	67.50 ± 3.8 a	78.88 ± 4.7 ab	94.25 ± 6.7 ab	103.50 ± 1.9 ab	111.38 ± 1.5 a
DHA 10 mM + PA 300 µM	21.25 ± 0.7 ab	33.25 ± 0.8 a	50.31 ± 1.5 a	61.63 ± 1.6 ab	68.56 ± 1.5 a	82.25 ± 1.5 ab	96.50 ± 2.5 ab	106.50 ± 1.9 ab	116.00 ± 1.3 a
DMSO 300 µM	22.50 ± 0.8 a	30.63 ± 2.1 a	46.19 ± 2.4 a	53.88 ± 3.4 b	64.81 ± 2.7 a	76.00 ± 3.2 ab	93.13 ± 1.8 ab	102.63 ± 5.2 ab	108.75 ± 5.2 a

The values represented are the heights (mean ± SE, n = 8) recorded at 10 day intervals during the entire growth of the plants. Different letters within a column indicate a statistically significant difference (DMRT; α = 0.05).

**Table 2:** Effect of DHA and PA on rice cv. BRRIdhan 28 growth and yield parameters recorded at the time of harvest (120 days after transplanting). The experiment was conducted in pots in a net house during the Boro season (January – April 2022). Plants were treated with DHA or PA alone or in combination as a foliar application or mock-treated with water or DMSO 15 days after transplanting (DAT) into the pots, followed by six spraying at 10 days intervals

Treatments	Tillers/hill	Effective tillers/hill	Panicle length (cm)	1000 grain weight (g)	Total grains/ panicle	Healthy grains/panicle	Unfilled/diseased grains/panicle	Straw yield (g)
Control	31.25 ± 2.8 b	21.38 ± 1.8 a	36.44 ± 0.8 b	23.80 ± 0.7 b	114.88 ± 11.3 c	22.13 ± 13.8 b	92.75 ± 9.8 a	40.10 ± 2.4 a
DHA 10 mM	37.63 ± 3.4 ab	26.88 ± 3.0 a	46.25 ± 2.9 a	25.93 ± 1.2 a	118.50 ± 6.5 c	87.00 ± 7.1 a	31.75 ± 4.8 c	39.65 ± 2.1 a
DHA 20 mM	42.25 ± 2.8 a	28.00 ± 2.4 a	47.00 ± 1.5 a	26.10 ± 1.0 a	172.88 ± 11.2 a	100.50 ± 7.8 a	72.38 ± ab	45.22 ± 5.0 a
PA 300 µM	35.25 ± 2.2 ab	24.38 ± 2.4 a	44.63 ± 1.7 a	24.11 ± 0.8 a	125.00 ± 17.3 bc	79.25 ± 7.9 a	45.75 ± 10.6 bc	38.13 ± 2.4 a
DHA 10 mM + PA 300 µM	39.88 ± 3.6 ab	27.63 ± 3.1 a	46.75 ± 1.7 a	26.38 ± 0.9 a	157.25 ± 12.1 ab	87.63 ± 9.2 a	70.38 ± 10.4 ab	40.56 ± 1.9 a
DMSO 300 µM	33.38 ± 3.1 ab	22.25 ± 2.4 a	42.57 ± 2.3 a	10.59 ± 4.1 a	148.38 ± 11.2 abc	77.38 ± 5.9 a	71.00 ± 9.1 ab	37.19 ± 2.4 a

The values represented are the heights (mean ± SE, n = 8) recorded at 10 day intervals during the entire growth of the plants. Different letters within a column indicate a statistically significant difference (DMRT; α = 0.05).

The pot study was repeated during the Aman season (August - November 2022) using cultivar BRRIdhan 49. Similar to the pot study in Boro season, a strong reduction in galls was observed upon repeated application of 10 or 20 mM DHA, 300 µM PA, and in the combined application of DHA 10 mM and PA 300 µM (Fig. 3) with a corresponding increase in yield. Similarly, an overall increase in plant growth and yield contributing characters was observed in these treatments (Tables 3 and 4). These results confirm the observations of the pot study conducted in Boro season (January - April 2022) and show that DHA is also effective in reducing nematode infection in cultivar BRRIdhan 49.

**Table 3:** Effect of DHA and PA on growth of rice cv. BRRIdhan 49. The experiment was conducted in pots in a net house during the Aman season (August – November 2022). Plants were treated with DHA or PA alone or in combination as a foliar application or mock-treated with water or DMSO 15 days after transplanting (DAT) into the pots, followed by six spraying at 10 days intervals

Treatments	15 DAT	25 DAT	35 DAT	45 DAT	55 DAT	65 DAT	75 DAT	120 DAT
Control	42.63 ± 3.5 a	54.31 ± 4.9 a	67.44 ± 4.9 a	75.06 ± 2.8 c	78.19 ± 2.6 b	81.13 ± 2.1 a	86.38 ± 1.5 b	88.38 ± 1.3 c
DHA 10 mM	41.81 ± 2.3 a	57.81 ± 3.6 a	74.00 ± 1.1 ab	81.31 ± 1.1 ab	82.44 ± 0.8 ab	84.75 ± 0.5 a	91.25 ± 1.2 a	95.25 ± 2.2 ab
DHA 20 mM	45.81 ± 1.7 a	63.38 ± 0.9 a	76.88 ± 1.8 a	82.56 ± 0.6 a	85.38 ± 1.0 a	85.50 ± 1.1 a	92.00 ± 0.8 a	101.25 ± 2.8 a
PA 300 µM	41.25 ± 3.4 a	56.44 ± 5.5 a	71.56 ± 2.9 ab	80.19 ± 0.9 ab	81.38 ± 0.7 ab	84.13 ± 0.4 a	88.63 ± 1.0 ab	88.63 ± 1.0 c
DHA 10 mM + PA 300 µM	43.06 ± 1.4 a	58.56 ± 2.1 a	75.25 ± 0.6 ab	81.44 ± 0.7 ab	84.06 ± 0.7 ab	85.13 ± 0.9 a	91.63 ± 0.9 a	97.63 ± 2.0 a
DMSO 300 µM	43.19 ± 2.4 a	56.50 ± 2.7 a	70.50 ± 1.9 ab	76.25 ± 2.5 bc	80.38 ± 2.5 ab	81.94 ± 2.5 a	86.13 ± 2.7 b	91.38 ± 2.9 bc

The values represented are the mean ± SE from eight plants. Different letters within a column indicate a statistically significant difference (DMRT; α = 0.05).

**Table 4:** Effect of DHA and PA on rice growth and yield parameters recorded at the time of harvest (120 days after transplanting). The experiment was conducted in pots in a net house during the Aman season (August – November 2022). Plants were treated with DHA or PA alone or in combination as a foliar application or mock-treated with water or DMSO 15 days after transplanting (DAT) into the pots, followed by six spraying at 10 days intervals

Treatments	Tillers/hill	Effective tillers/hill	Panicle length (cm)	1000 grain weight (g)	Total grains/panicle	Healthy grains/panicle	Unfilled/diseased grains/panicle	Straw yield (g)
Control	36.75 ± 2.3 c	25.00 ± 1.9 c	38.47 ± 1.2 d	15.71 ± 0.6 b	125.38 ± 10.3 d	73.38 ± 8.9 e	52.00 ± 5.3 a	35.75 ± 3.4 d
DHA 10 mM	51.63 ± 2.6 ab	33.25 ± 0.8 b	47.13 ± 0.6 b	16.21 ± 0.5 b	154.00 ± 5.0 bc	135.25 ± 5.0 bc	18.75 ± 2.0 c	58.71 ± 1.1 c
DHA 20 mM	58.00 ± 1.8 a	46.13 ± 1.5 a	51.50 ± 0.4 a	22.96 ± 1.8 a	180.00 ± 5.4 a	171.75 ± 5.2 a	8.25 ± 2.0 d	89.87 ± 2.7 a
PA 300 µM	48.63 ± 4.1 b	29.88 ± 2.8 bc	43.50 ± 0.8 c	16.00 ± 0.4 b	149.50 ± 5.9 bc	125.38 ± 5.4 c	24.13 ± 1.4 c	51.90 ± 1.3 c
DHA 10 mM + PA 300 µM	55.13 ± 3.1 ab	42.63 ± 1.6 a	48.06 ± 0.4 b	17.24 ± 0.4 b	166.25 ± 6.7 ab	150.75 ± 6.9 b	15.50 ± 0.8 cd	68.91 ± 2.0 b
DMSO 300 µM	38.88 ± 2.6 c	27.63 ± 0.8 c	39.38 ± 1.9 d	15.46 ± 0.5 b	139.50 ± 7.2 cd	102.13 ± 5.8 d	37.38 ± 5.1 b	34.11 ± 3.5 d

The values represented are the mean ± SE of eight plants. Different letters within a column indicate a statistically significant difference (DMRT; α = 0.05).

### Field Efficacy of DHA

**Fig. 4:**
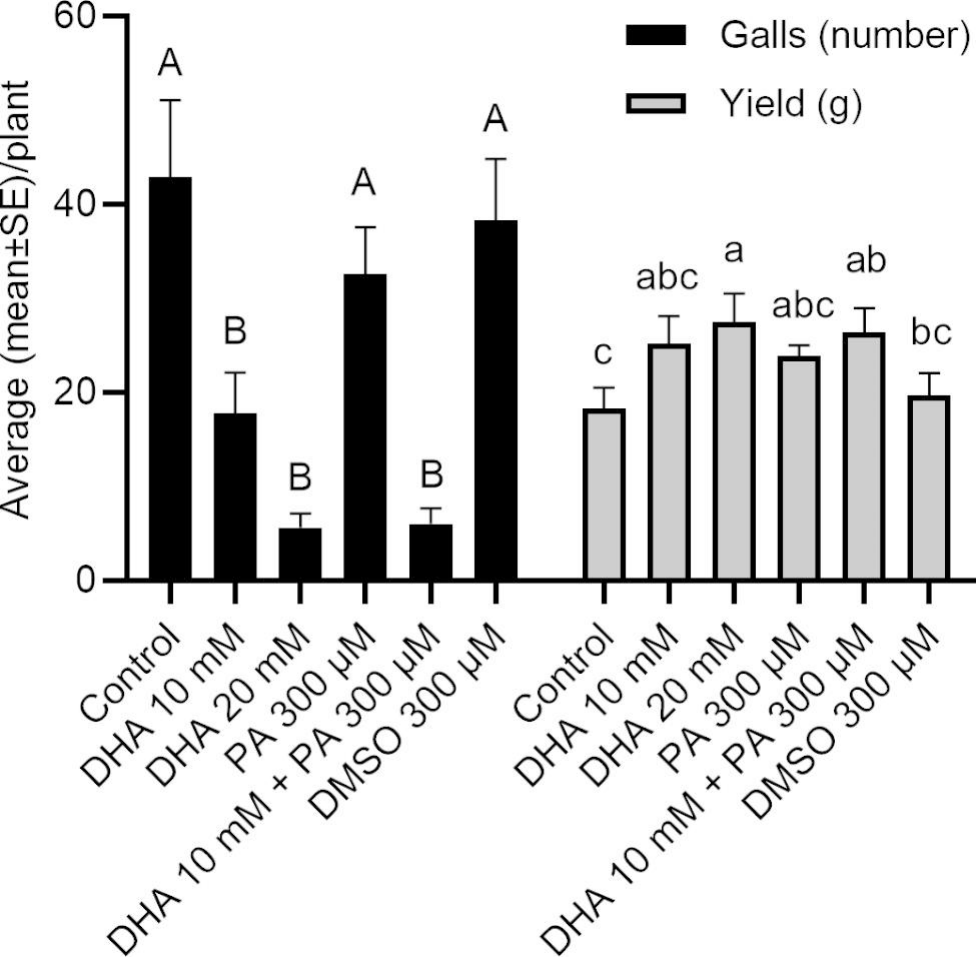
Field efficacy of DHA and PA in rice against *Meloidogyne graminicola*. The experiment was conducted in a naturally nematode-infested field. Plants were treated with DHA or PA alone or in combination as a foliar application or mock-treated with water or DMSO 15 days after transplanting (DAT) to the main field, followed by six spraying at 10 days intervals. Effect on galls and seed yield was recorded at the time of harvesting (120 DAT). Bars on each column indicate SE from eight replicates. Different letters on error bars within a group indicate a statistically significant difference (DMRT; α = 0.05)

The field efficacy of DHA and PA alone or in combination was evaluated against *M. graminicola* in a naturally nematode-infested field. The initial nematode population (Pi) was 800 J2s/100 g of soil, confirming significant infestation of this field. Confirming lab and net-house experiments, repeated foliar application of DHA 10 or 20 mM, and a combination of DHA 10 mM + PA 300 µM led to significantly lower nematode infection (Fig. 4). The number of galls varied from 6 to 43 per plant among the treatments (Fig. 4). The strongest reduction in galls was observed when plants were regularly treated with DHA 20 mM (87% reduction compared to untreated control), followed by DHA 10 mM + PA 300 µM (86% reduction compared to untreated control). Corresponding increase in seed yield was observed upon these treatments (Fig. 4). The maximum yield was recorded in plants treated with DHA 20 mM (27.46 g) followed by DHA 10 mM + PA 300 µM combination (26.44 g). The lowest seed yield was recorded in the untreated control plants (18.33 g), followed by plants treated with the second control treatment: 300 µM DMSO (the solvent for PA). Treatments with DHA alone or in combination with PA has no detectable negative effects on plant growth (Tables 5 and 6). The best plant growth was observed in plants treated with DHA alone or in combination with PA (Table 5). Our results revealed that the number of tillers, panicles, number of grains per panicle, panicle length, total grains per panicle, filled/healthy grains per panicle, and dry matter accumulation were significantly higher upon repeated foliar DHA 20 mM or DHA 10 mM + PA 300 µM treatment (Table 6). These results show that DHA alone or in combination with PA significantly reduces nematode infection and increases seed yield in rice.

**Table 5:** Field efficacy of DHA and PA on rice growth throughout the season. The experiment was conducted in a naturally nematode-infested field. Plants were treated with DHA or PA alone or in combination as a foliar application or mock-treated with water or DMSO 15 days after transplanting (DAT) to the main field, followed by six spraying at 10 days intervals

Treatments	15 DAT	25 DAT	35 DAT	45 DAT	55 DAT	65 DAT	75 DAT	85 DAT	120 DAT
Control	22.69 ± 1.4 c	27.56 ± 1.2 b	30.00 ± 1.6 b	35.50 ± 1.3 c	40.88 ±1.5 c	49.63 ±1.3 c	55.50 ± 1.7 c	67.86 ± 3.1 d	80.00 ± 2.2 c
DHA 10 mM	25.38 ± 2.0 abc	30.75 ± 1.5 ab	31.38 ± 1.5 b	40.06 ± 1.2 b	45.88 ± 1.6 bc	59.06 ± 2.8 ab	65.69 ± 3.1 b	81.32 ± 1.3 ab	86.50 ± 1.8 ab
DHA 20 mM	26.94 ± 0.9 a	32.21 ± 0.8 a	34.94 ± 1.3 a	45.31 ± 1.2 a	51.94 ± 1.6 a	61.81 ± 2.7 a	73.68 ± 2.7 a	86.69 ± 1.7 a	89.50 ± 1.2 a
PA 300 µM	23.06 ± 1.0 bc	28.53 ± 0.6 b	30.38 ± 0.7 b	37.0 ± 1.0 bc	43.73 ± 1.8 bc	53.75 ± 1.7 bc	65.30 ± 1.6 b	78.75 ± 1.7 bc	82.13 ± 2.3 bc
DHA 10 mM + PA 300 µM	26.13 ± 1.0 ab	30.63 ± 1.0 ab	33.25 ± 0.8 ab	40.06 ± 0.7 b	48.45 ± 0.6 ab	60.63 ± 1.0 a	73.50 ± 1.6 a	84.06 ± 2.3 ab	88.50 ± 1.8 a
DMSO 300 µM	22.13 ± 0.7 bc	27.75 ± 0.6 b	30.50 ± 0.9 b	36.40 ± 2.0 bc	43.09 ± 2.3 c	49.63 ± 1.7 c	63.86 ± 2.7 b	73.25 ± 3.1 cd	80.63 ± 2.0 c

The values represented are the heights (mean ± SE, n = 8) recorded at 10 day intervals during the entire growth of the plants. Different letters within a column indicate a statistically significant difference (DMRT; α = 0.05).

**Table 6:** Field efficacy of DHA and PA on yield attributes of rice recorded at the time of harvest (120 days after transplanting). The experiment was conducted in a naturally nematode-infested field. Plants were treated with DHA or PA alone or in combination as a foliar application or mock-treated with water or DMSO 15 days after transplanting (DAT) to the main field, followed by six spraying at 10 days intervals

Treatments	Tillers/hill	Effective tillers/hill	Panicle length (cm)	1000 grain weight (g)	Total grains/ panicle	Healthy grains/panicle	Unfilled/diseased grains/ panicle	Straw yield (g)
Control	18.75 ± 1.6 b	11.38 ± 0.6 b	38.61 ± 1.1 b	32.59 ± 0.3 a	74.63 ± 5.9 b	69.62 ± 5.9 c	5.00 ± 1.2 bc	17.72 ± 1.8 d
DHA 10 mM	19.88 ± 1.3 b	14.00 ± 1.1 ab	41.61 ± 2.1 ab	34.51 ± 0.9 a	89.38 ± 5.7 a	76.13 ± 7.8 bc	13.25 ± 5.0 a	25.53 ± 2.4 ab
DHA 20 mM	25.63 ± 1.5 a	16.00 ± 1.7 a	43.73 ± 0.9 a	34.86 ± 0.9 a	102.50 ± 5.3 ab	98.00 ± 5.2 a	4.50 ± 0.5 bc	27.61 ± 1.1 a
PA 300 µM	19.63 ± 1.6 b	13.50 ± 1.4 ab	40.99 ± 1.5 ab	34.34 ± 0.8 a	88.50 ± 4.7 a	76.75 ± 8.0 bc	11.75 ± 3.5 ab	22.59 ± 1.0 bc
DHA 10 mM + PA 300 µM	24.13 ± 0.9 a	15.75 ± 1.2 a	42.23 ± 1.3 ab	34.69 ± 0.8 a	96.13 ± 6.1 ab	91.38 ± 6.0 ab	4.75 ± 1.0 bc	26.55 ± 1.4 ab
DMSO 300 µM	19.00 ± 1.7 b	12.25 ± 1.1 ab	41.26 ± 1.6 ab	33.15 ± 1.2 a	76.75 ± 5.9 b	74.00 ± 5.8 bc	2.75 ± 0.3 c	19.18 ± 1.7 cd

The values represented are the mean ± SE of eight plants. Different letters within a column indicate a statistically significant difference (DMRT; α = 0.05).

### DHA is Nematicidal to the Second-stage Juveniles of ***M. graminicola***

**Fig. 5:**
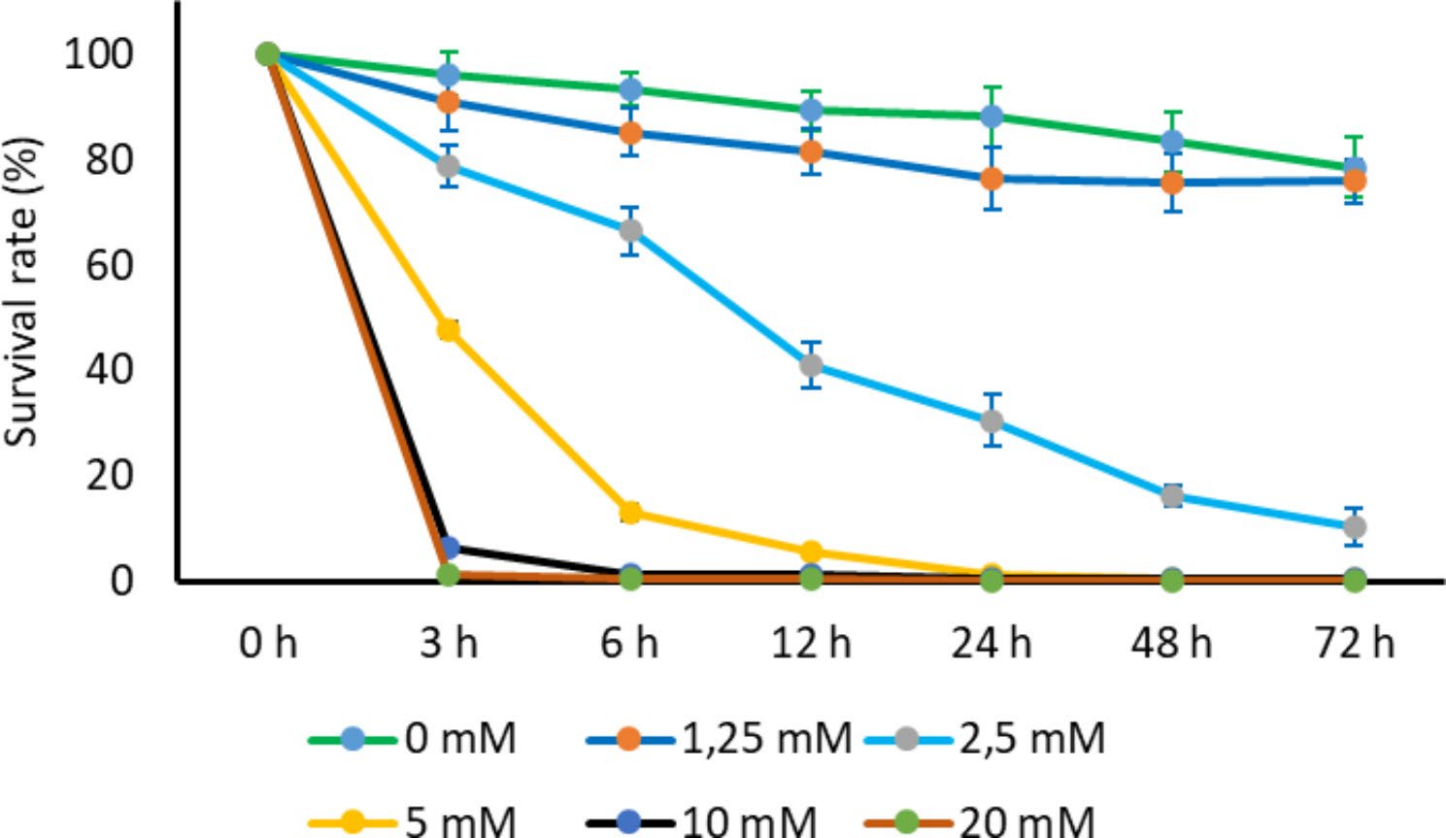
The percent nematode survival of second-stage juveniles (J2s) of *Meloidogyne graminicola* upon direct exposure to DHA. Around 100 freshly hatched J2s were incubated in solutions containing different concentrations of DHA: 0 mM, 1.25 mM, 2.5 mM, 5 mM, 10 mM and 20 mM. Each treatment was replicated four times. Observations on nematode mortality were recorded 3, 6, 12, 24, 48, and 72 h after incubation. The experiment was independently repeated two times, providing confirmatory results

Previously, we demonstrated that foliar DHA application leads to the induction of systemic resistance, by activation of plant SA and ROS signaling (Chavan et al. 2022). However, it was unclear if DHA could also have direct effects on the nematodes. In an in vitro bioassay, DHA caused strong mortality to the J2s of *M. graminicola* (Fig. 5). Among the different concentrations evaluated, DHA was found nematicidal at concentrations ranging from 2.5 to 20 mM (Fig. 5). A clear dose-response effect was observed and more than 90% nematode mortality was observed within 3 h of exposure to 10 and 20 mM concentrations. These results indicate a quick nematoxic effect of DHA.

A low LC_50_ of 4.95 to 2.74 mM was observed at 6 to 72 h time points (Table 7), which indicates very high nematode mortality. These data indicate that DHA is highly toxic to the J2s of *M. graminicola*.

### The Efficacy of DHA can be Improved Using Different Methods of Application

**Fig. 6:**
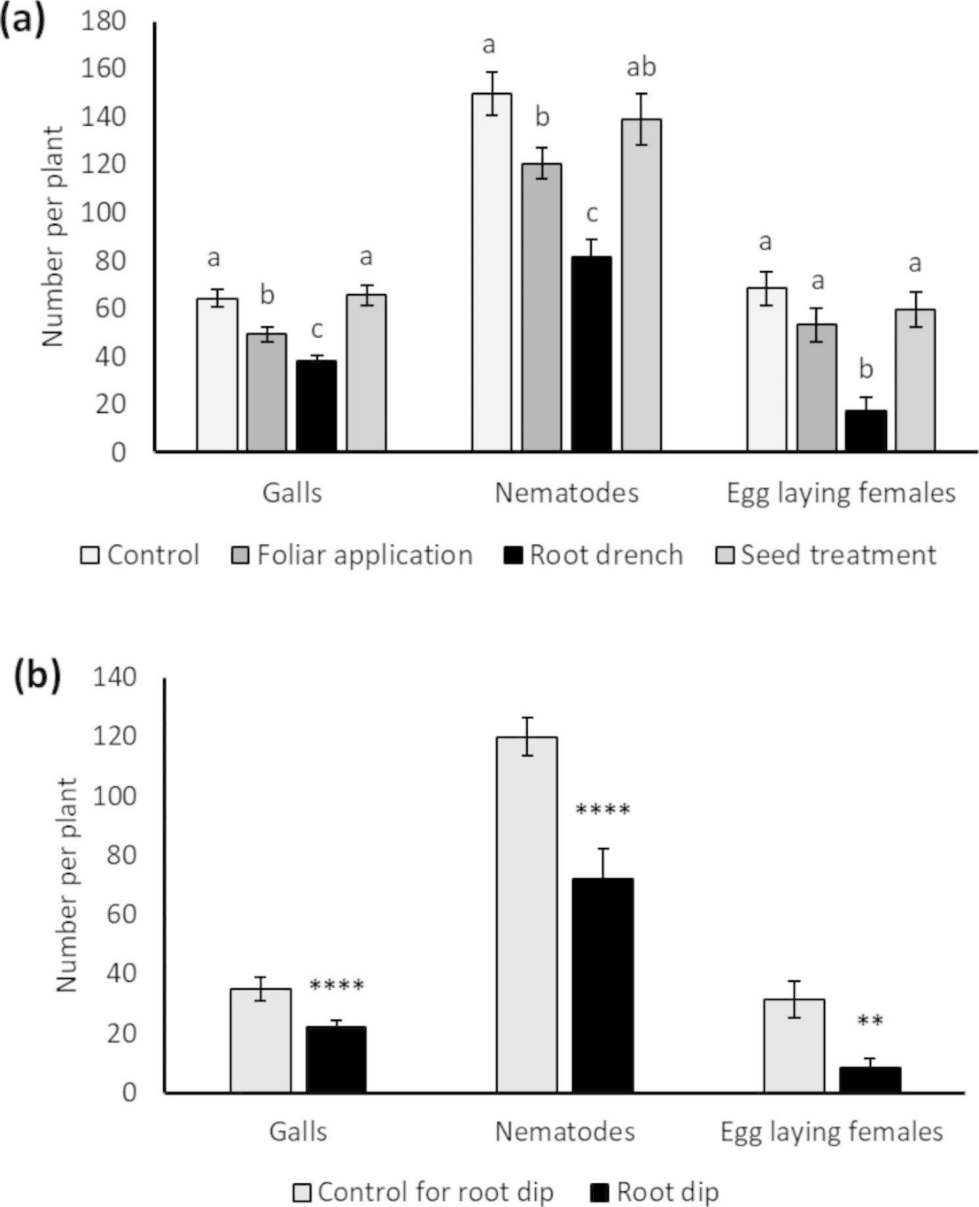
Evaluation of different methods of dehydroascorbate (DHA) application in rice. Effect of DHA application as (**a**) foliar application, root drench, seed treatment (**b**) root dip application in rice against *M. graminicola*. Plants were treated with 20 mM DHA followed by nematode inoculation of 250 J2 per plant 24 h post-treatment of foliar application, root drench, and root dip, while two weeks post planting of seedlings obtained from seed treatment. Observations on galls, total nematodes, and egg-laying females were recorded two weeks post nematode inoculation using the acid fuchsin staining technique. Error bars on each column represent the SE from 16 replications. Different letters on error bars within a group in (**a**) indicate a statistically significant difference (DMRT; α = 0.05). *Asterisks on error bars in (**b**) indicate statistically significant difference (Student’s t-test, * = p < 0.05, ** = p < 0.01)

Based on the observed dual action of DHA, being both IR-stimulating (Chavan et al. 2022) and nematoxic (Fig. 5), we decided to evaluate if - next to foliar spraying - other application methods could enhance its efficacy. In a lab experiment, different methods of 20 mM DHA application were compared: foliar treatment, root drench, root dip, and seed treatment. Except for seed treatment, all other methods were found effective in significantly reducing the rice susceptibility to *M. graminicola* (Fig. 6). The highest reduction in galls and nematodes was observed using a root drench (40 and 45%) or root dip method (37 and 39%) followed by foliar treatment (23 and 20%) (Fig. 6) compared to the mock-treated control. This increased efficacy when using root application could be explained by the nematoxic effect of DHA on the root-feeding nematodes.

## Discussion

Resistance inducers hold great potential for integrated pest management but remain rarely used due to concerns about potential yield penalties and limited efficacy (Walters and Fountaine 2009; Walters et al. 2013; Yassin et al. 2021). However, they can be combined with other compatible IPM techniques to improve efficacy and remediate potential adverse effects (Yassin et al. 2021). Our work demonstrates that DHA is an effective novel nematode control strategy based on a dual mode-of-action: it induces resistance in rice against *M. graminicola*, and has nematicidal properties. Interestingly, spray application of this compound does not cause negative effects on plant growth and yield. Our data demonstrate that its efficacy can be improved by combining it with other IR stimulants such as PA or by using different methods of application.

The resistance induced by several IR stimuli has been suggested to be long-lasting, although the longevity might vary depending on the compound (Luna et al. 2014; Walters et al. 2013). Foliar pretreatment of rice plants with DHA induces plant defence at 1 DPT (Chavan et al. 2022) and has significant negative effects on the number of galls, nematodes and egg-laying females detected at 14 days after inoculation in lab studies (Figs. 1 and 2a; Chavan et al. 2022). A similar effect in reducing rice susceptibility to *M. graminicola* was observed on plants inoculated with nematodes up to 7 days post DHA treatment (Fig. 2c), indicating that rice plants retain the IR memory for a longer period. The IR effect seems to vanish at 14 DPT, where only a reduction in number of nematodes was observed (Fig. 2d). These results suggest that repeated application of DHA is essential at or before the 14-day interval. In line with these results, N-3-oxo-tetradecanoyl-L-homoserine lactone (oxo-C14-HSL) treatment in soyabean led to the long-term priming effect against root-lesion nematode *Pratylenchus penetrans* (Adss et al. 2021). Reduced root lesions were observed in the oxo-C14-HSL-treated soybean plants compared to non-treated plants when the nematodes were added 3, 7, or 15 days later. Similarly, BABA-IR in *Arabidopsis* could be detected up to 28 days after treatment (Luna et al. 2014). Based on our observations that the IR-effect vanishes at 14 DPT, a 10-day interval between the applications was chosen for the pot and field experiments.

Although IR might provide long-lasting protection, in terms of practical disease control, the frequency of application is a crucial consideration (Walters et al. 2013). For example, multiple spray treatments with 2000 mg/L BABA at 10-day intervals significantly reduced the number of *Heterodera avenae* cysts on wheat and barley (Oka and Cohen 2001). Similarly, multiple pre-harvest treatments with ASM induce resistance in muskmelon and reduce latent infection in fruits caused by *Alternaria alternata* and *Fusarium* spp. (Zhang et al. 2011). The authors further showed detectable increases in defence-related enzyme activities and metabolite levels in plants upon repeated ASM treatments. Similarly, in field experiments examining the efficacy of ASM against the bacterial spot on tomato, weekly applications provided considerably better disease control than applications every two weeks (Huang et al. 2012). Similar to these reports, our study revealed that a repeated DHA treatment at a 10-day interval strongly reduced nematode infection compared to untreated control plants in pot and field experiments (Figs. 3 and 4).

Field application of IR agents often shows a lack of consistency and incomplete disease control (Walters and Fountaine 2009). As IR is a host response, its expression under field conditions is influenced by several factors, including the environment, genotype, crop nutrition, and the extent to which plants are already induced by other factors (Walters et al. 2013). Our pot and field studies revealed that both DHA alone or in combination with PA was effective in reducing *M. graminicola* infection with a corresponding increase in rice yield in 2 popular Bangladeshi rice cultivars (Figs. 3 and 4). Similar to our results, BTH and SA were shown to activate IR in faba bean against rust (*Uromyces viciae-fabae*) and ascochyta blight (*Ascochyta fabae*) under both glasshouse and field conditions (Sillero et al. 2012).Foliar application of BTH also provided protection against the root-infecting parasitic *Orobanche crenata* on pea (Pérez-de-Luque et al. 2004) and faba bean (Sillero et al. 2012). Similarly, (Desmedt et al. 2021) evaluated PA application in greenhouse-grown tomato naturally infested with *M. incognita* and *M. javanica*. A significant reduction in gall index was observed in PA-treated plants compared to untreated plants.

The efficacy of IR inducers can be improved by combining them with other compatible techniques (Yassin et al. 2021). The use of low doses of multiple agents for additive or synergistic IR effects is a potential means of improving their efficacy (Yassin et al. 2021). Our results confirm that DHA can be combined with another IR stimulus, PA (Figs. 3 and 4) and that the dose of DHA can be reduced in this combination treatment (Figs. 3 and 4). The combined application of BABA-BTH at half the recommended dose had an additive effect in effectively controlling *Plasmopara viticola* in grapevines (Reuveni et al. 2001). Similarly, Walters et al. (2011) reported improved control of powdery mildew in barley using combined treatments of ASM, BABA and JA. Although used at different doses, DHA and PA induce similar kinds of host IR responses, such as the activation of ROS metabolism, SA, and the diterpenoid phytoalexin pathway (Chavan et al. 2022; Desmedt et al. 2021, 2022b). Desmedt et al. (2022a) showed that distinct IR stimuli viz., BABA, ASM, DHA, and PA, capable of inducing systemic IR in rice against the RKN *M. graminicola*, share common transcriptional responses such as the induction of JA and phenylpropanoid pathway metabolism in the systemic tissues. The compatibility of DHA and PA shows great scope for this combination treatment to evaluate for a broad spectrum of stresses in rice.

Identifying compounds combining biocidal and IR activity could improve control efficacy (Yassin et al. 2021). In an effort to find such dual-action compounds, Schillheim et al. (2017) developed a high-throughput assay to screen cultured parsley for compounds that prime the secretion of antimicrobial phytoalexins and reported 1-isothiocyanato-4-methylsulfinylbutane (sulforaphane, SFN) with dual mode of action. SFN primed *WRKY6* gene expression in *Arabidopsis* and reduced susceptibility to *Hyaloperonospora arabidopsidis*. Additionally, It showed broad antimicrobial action against oomycete *H. arabidopsidis*, fungus *Plectosphaerella cucumerina*, and bacterium *Pseudomonas syringae*. Similarly, BABA-induced protection of *Brassica napus* against fungal pathogen *Leptosphaeria maculans* was associated with a combination of modes of action, as it induced SA synthesis and (pathogenesis-related) *PR-1* expression, in addition to the fungitoxic effect against *L. maculans* (Šašek et al., 2012). Similarly, Schouteden et al. (2017) reported the direct nematicidal property of classical IR agents MeJA and ASM against RKN *M. incognita*. Our results revealed that next to activation of induced systemic resistance (Figs. 1 and 2), DHA also causes strong direct dose-dependent mortality to the J2s of *M. graminicola* with an LC_50_ of 4.95 to 2.74 mM (Fig. 5 and Table 7). In line with these results, the melon Cold Peeling Extract (mCOPE) - activating IR against RKN in rice and tomato - was also found nematicidal to the J2s of *M. graminicola* and *M. incognita* (De Kesel et al. 2022). mCOPE caused strong nematode mortality (about 100%) to the J2s of *M. graminicola* and *M. incognita* within 24 h of exposure (De Kesel et al. 2022).

**Table 7:** Probit analysis results of dehydroascorbate (DHA) against second-stage juveniles (J2s) of *Meloidogyne graminicola*. Around 400 freshly hatched J2s were exposed to different concentrations of DHA: 1, 2, 3, 4, 5, and 6 mM. The observations on nematode mortality were recorded 6, 12, 24, 48, and 72 h after exposure

Time points	n	Slope (± SE)	LC_50_ (mM) (95% CL)	χ2 goodness-of-fit (P value)
6 h	400	8.50 (0.49)	4.95 (4.15–5.92)	41.55 (0.000)
12 h	400	6.81 (0.33)	4.27 (3.72–4.79)	23.63 (0.000)
24 h	400	6.69 (0.27)	3.50 (3.09–3.87)	18.62 (0.000)
48 h	400	5.92 (0.25)	2.97 (1.53–3.83)	87.72 (0.000)
72 h	400	6.46 (0.27)	2.74 (1.50–3.93)	156.28 (0.000)

n = Number of individuals (J2s) included in the analysis.

IR activators at concentrations suitable for different plant growth stages and applied by the proper method can possibly be included in IPM programs for nematode management (Molinari 2016; Pankaj et al. 2013). Soil drenches with SA and INA (2,6-dichloroisonicotinic acid) and root dip application of SA and BTH inhibited RKN reproduction, at specific dose ranges, without affecting plant growth in tomato, brinjal, and pepper (Molinari 2016). Similarly, in evaluating different methods of DHA application in rice, foliar application, soil drench, and root dip methods were found to be significantly effective in reducing rice susceptibility to *M. graminicola* (Fig. 6). However, DHA was ineffective when used as a seed treatment (Fig. 6a). In contrast, seed treatment with other IR activators like BABA was significantly effective in suppressing *M. javanica* infection in tomato (Fatemy et al. 2012). Similarly, jasmonic acid (JA) seed treatment was effective against RKN in cowpea and tomato and cyst nematodes in potato (Pankaj et al. 2013). The non-effectiveness of DHA as a seed treatment may be because DHA is not stable for a longer time (Huelin 1949) or because seeds might not have absorbed DHA sufficiently. Alternatively, the longevity of DHA-IR in seed treatment might have vanished at the time of nematode inoculation (14 days post-germination). However, evaluating DHA as a seed treatment in naturally nematode infested nursery beds can be interesting for future studies, since DHA is also nematicidal to *M. graminicola*. An increased effectiveness was observed when DHA was applied as a soil drench and root dip method compared to the foliar application (Fig. 6). This increased efficacy is likely explained by the dual action of DHA acting as both nematicide and IR-stimulus (Figs. 1 and 5).

Activation of plant defence has been described to be associated with a fitness cost, as it requires energy and resources (Walters et al. 2013). However, the extent of the fitness penalty differs largely between stimuli and is dependent on the growth environment (Van Hulten et al. 2006; Walters and Heil 2007). Hence, potential changes in plant growth should be monitored upon IR activation (Yassin et al. 2021). DHA did not cause any negative effects on the plant growth of rice plants (Figs. 1b and 3, and 4). Interestingly, significantly increased panicle length and number, tiller number and overall seed yield was observed in DHA-treated or DHA + PA-treated rice plants (Figs. 3 and 4; Tables 1, 3, 2, 4, 5 and 6), making these treatments suitable for use in crop protection. The increase in growth and yield in these treated plants may be because of the reduction in nematode infection and plant growth promotion by DHA. A significant accumulation of auxin indole-3-acetic acid upon DHA treatment (Chavan et al. 2022) suggests that the increased production of growth hormones might balance the induction of defence in DHA-IR and as such avoid fitness costs. Moreover, several reports have highlighted the role of DHA in promoting cell growth and division (Horemans et al. 2003; Potters et al. 2002; Tyburski et al. 2008).

## Conclusion

Collectively, our lab, pot, and field experiments show the potential of DHA as a control strategy for the effective management of *M. graminicola* in rice. Due to its dual action as both nematicide and IR-stimulus, DHA can be utilized effectively for the management of nematode problems in crops. While further ecotoxicological assessments will be required before DHA can be utilized for practical use, overall, our results indicate the potential of DHA as a sustainable crop protection product.

## Electronic Supplementary Material

Below is the link to the electronic supplementary material.

**Additional File 1: Supplementary Figure S1**. Experimental set up of lab nematode infection. **Supplementary Figure S2**. Experimental set up of pot study. **Supplementary Figure S3**. Experimental set up of field study. **Supplementary Figure S4**. In vitro bioassay showing (a) live (healthy and moving) and (b) dead nematodes (second-stage juveniles, J2s of *Meloidogyne graminicola*) from control and DHA solutions, respectively. **Supplementary Figure S5**. Effect of dehydroascorbate (DHA) on rice susceptibility to *Meloidogyne graminicola*.

## Data Availability

The data that support the findings of this study are available within the paper, and more information, if required, can be requested to the corresponding author.

## Abbreviations

DHA: Dehydroascorbate
DAT: Days after transplanting
DPT: Days post-treatment
DPI: Days post inoculation
IR: Induced resistance
LC_50_: Median lethal concentration
J2s: Second-stage juveniles
PA: Piperonylic acid
