# Supplementary material for: Potential of Exogenous Treatment with Dehydroascorbate to Control Root-knot Nematode Infection in Rice
Source: Rice (N Y). 2023 Jun 29;16:29. doi: 10.1186/s12284-023-00644-1 (PMC10307936; doi:10.1186/s12284-023-00644-1)
Supplement: Supplementary file 1 — Additional File 1: Supplementary Figure S1. Experimental set up of lab nematode infection. Supplementary Figure S2. Experimental set up of pot study. Supplementary Figure S3. Experimental set up of field study. Supplementary Figure S4. In vitro bioassay showing (a) live (healthy and moving) and (b) dead nematodes (second-stage juveniles, J2s of Meloidogyne graminicola) from control and DHA solutions, respectively. Supplementary Figure S5. Effect of dehydroascorbate (DHA) on rice susceptibility to Meloidogyne graminicola. [file 12284_2023_644_MOESM1_ESM.pdf]

Title: Potential of exogenous treatment with dehydroascorbate to control root-knot nematode infection in rice

Authors: Satish Namdeo Chavan[a,b†], Farzana Haque Tumpa[a†], Md. Atiqur Rahman Khokon[c], Tina Kyndt[a]*

[a] Department of Biotechnology, Faculty of Bioscience Engineering, Ghent University, Proeftuinstraat 86 N1, Ghent-9000, Belgium

[b] ICAR-Indian Institute of Rice Research, Rajendranagar, Hyderabad 500030, India

[c] Department of Plant Pathology, Bangladesh Agricultural University, Mymensingh-2202, Bangladesh

* Correspondence: Tina Kyndt, Department of Biotechnology, Faculty of Bioscience Engineering, Ghent University, Ghent-9000, Belgium. Email: Tina.Kyndt@Ugent.be

Supplementary information

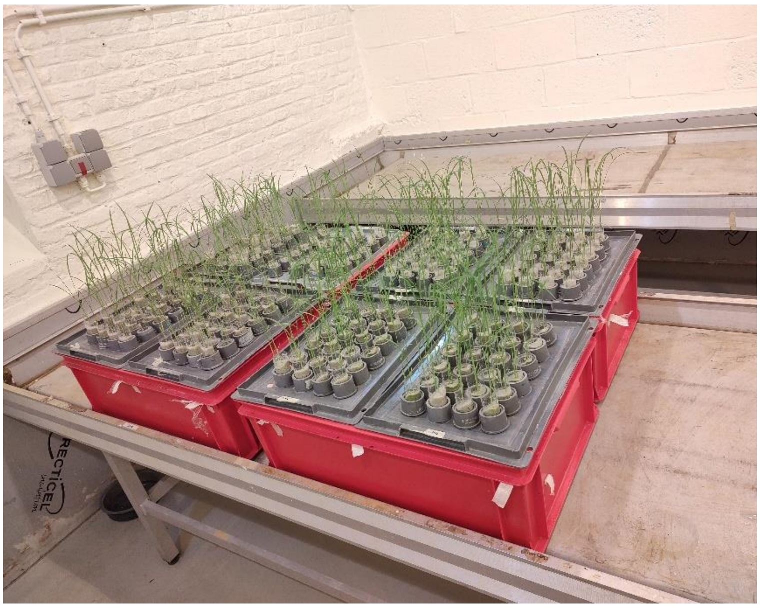

Supplementary Fig. S1 Experimental set up of lab nematode infection experiments performed in a rice growth room at the Department of Biotechnology, Faculty of Bioscience Engineering, Ghent University, Ghent, Belgium.

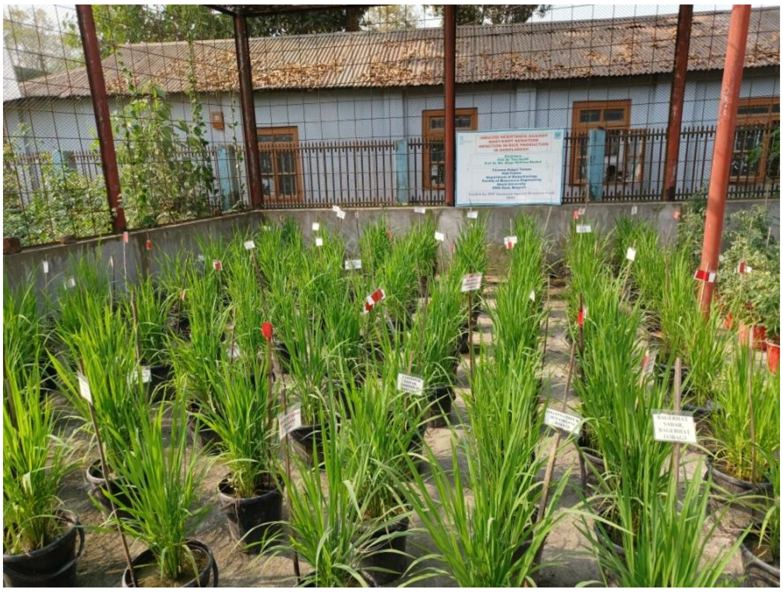

Supplementary Fig. S2 Experimental set up of pot study conducted in the net house of Professor Golam Ali Fakir Seed Pathology Centre, Department of Plant Pathology, Bangladesh Agricultural University, Mymensingh, Bangladesh.

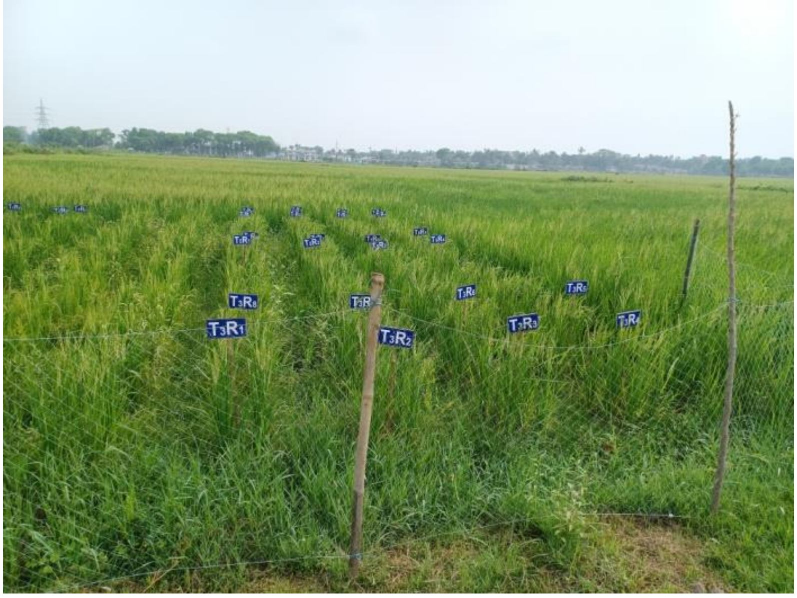

Supplementary Fig. S3 Experimental set up of field study conducted in a naturally nematode-infested field located at Central Farming System (CFS) research farm, Bangladesh Agriculture University, Mymensingh, Bangladesh.

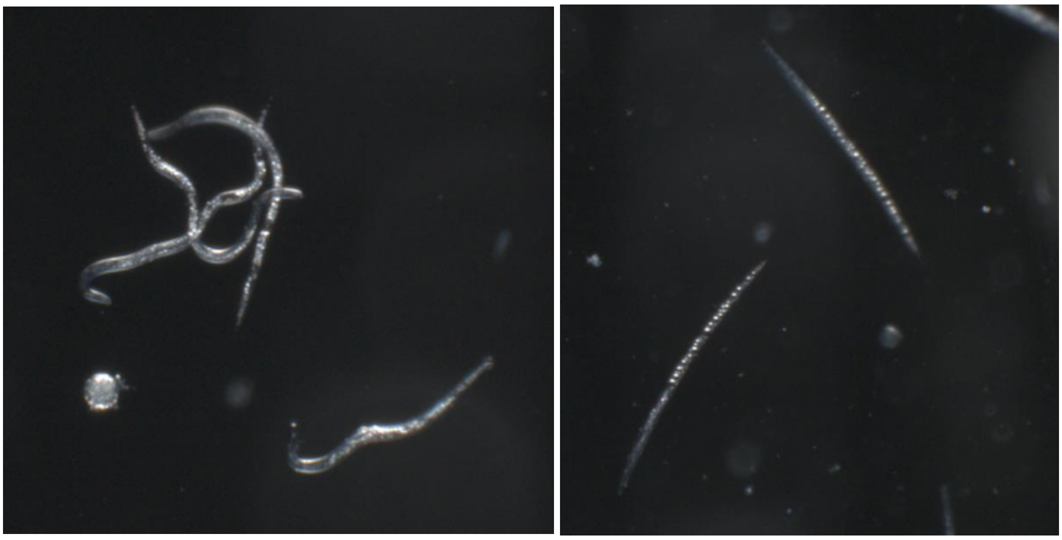

Supplementary Fig. S4 *In vitro* bioassay showing (a) live (healthy and moving) and (b) dead nematodes (second-stage juveniles, J2s of *Meloidogyne graminicola*) from control and DHA solutions, respectively. Images were obtained using a Leica microscope (S8AP0) connected to the digital camera (Leica DFC400 and Leica application suit 3.6).

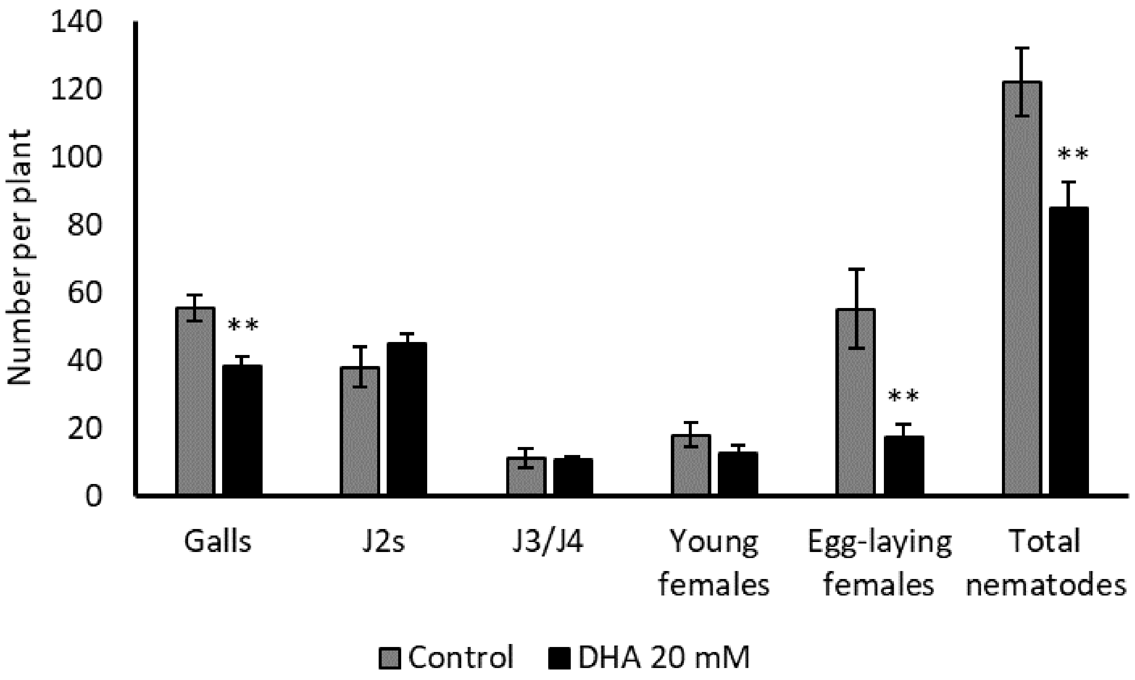

Supplementary Fig. S5 Effect of dehydroascorbate (DHA) on rice susceptibility to *Meloidogyne graminicola*. Two-week-old rice plants were treated with 20 mM DHA followed by nematode inoculation 250 J2s per plant 24 h post-treatment. Galls, different nematode developmental stages and total nematodes were recorded two-week post nematode inoculation. Bars on each column indicate SE from eight replicates. The experiment was independently repeated three times, providing confirmatory results. *Asterisks on error bar indicate statistically significant difference (Student's t-test, * = $p < 0.05$, ** = $p < 0.01$).
